# Thoracoscopic approach to the resection of idiopathic azygos vein aneurysm: a case report

**DOI:** 10.1186/s13019-022-01908-5

**Published:** 2022-06-20

**Authors:** Xuefeng Ling, Renjie Yu, Lei Fang, Xiaorong Zhang, Chuan Yao, Ketao Tu, Zhiying Chen

**Affiliations:** 1 Respiratory Medicine Department, Affiliated Hospital of Jiujiang University, Jiujiang, Jiangxi China; 2 Pathology Department, Affiliated Hospital of Jiujiang University, Jiujiang, Jiangxi China; 3 Cardio-Thoracic Surgical Department, Affiliated Hospital of Jiujiang University, Jiujiang, Jiangxi China; 4 Jiujiang Clinical Precision Medicine Research Center, Jiujiang, 332000 Jiangxi China

**Keywords:** Azygos veinaneurysm, Thoracoscopic surgery

## Abstract

**Background:**

Azygos vein aneurysm (AVA) is a rare thoracic pathological entity that mimics a posterior mediastinal mass. However, the pathogenesis of primary azygos vein aneurysms is not clear and its pathology is still being discussed. Some of the AVA are asymptomatic and usually discovered accidentally by routine physical examination.

**Case presentation:**

We report the case of a 37-year-old woman who had an azygos vein arch aneurysm with no obvious clinical symptoms. With the analysis of clinical features of the case and AVA morphological characteristics, the AVA was found by a chest computed tomography. Then, enhanced chest computed tomography showed a soft-tissue mass (4.9 × 3.7 × 3.2 cm) in the right posterior mediastinum, which was connected to the superior vena cava and significantly enhanced with contrast agent stratification. The density of the tumor in the delayed stage was the same as that in the azygos vein. The patient underwent video-assisted thoracoscopic surgery. Histopathological evaluation of the surgical biopsy specimen proved to be a completely thrombosed aneurism of the azygos vein arch.

**Conclusions:**

AVA is a rare pathology that must be taken into consideration during the differential diagnosis of right posterior mediastinal masses. Thoracoscopic surgery is one of the most preferred treatment options for azygos vein aneurysm.

**Supplementary Information:**

The online version contains supplementary material available at 10.1186/s13019-022-01908-5.

## Background

Azygos vein aneurysm is an extremely rare cause of the mediastinal mass that typically detected incidentally in asymptomatic patients by the chest radiograph or computed tomography [1]. In general, the preoperative diagnosis of saccular azygos vein aneurysms is defined by enhanced multidetector CT or magnetic resonance imaging [2–4]. Yet the AVA is parallel to other mediastinal masses such as neurogenic tumor, paratracheal and hilar lymph nodes, and bronchogenic cyst [5–8], so its dignosis is relatively difficult in cases with associated complete thrombosis. In the previously reported literature, cases of AVA clinical symptoms were presented as follows: back pain, chest tightness, dyspnea, and coughing and common complications such as thrombosis, rupture, or compression of adjacent organs have been described [8, 9]. The optimal treatment strategy for AVA remains uncertain. Current treatment options include conservative treatment [10], interventional treatment [11, 12], and surgical treatment [13, 14]. We report the case of a presentation of an idiopathic AVA, which was surgically resected by thoracoscopic approach. Futhermore, we discuss its aetiology, diagnostic methods and treatments.

### Case presentation

**Fig. 1:**
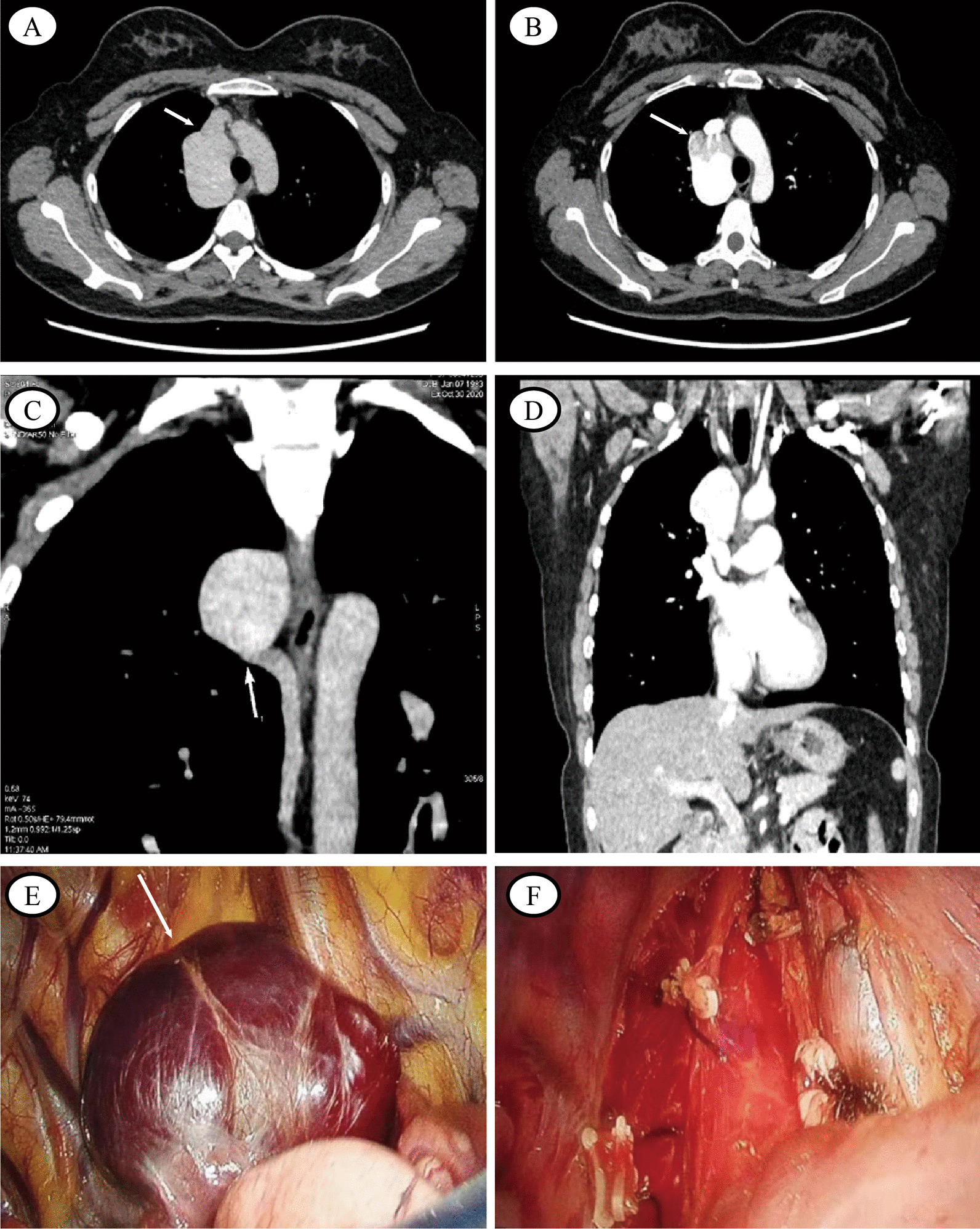
**A**, **B**: Chest computed tomography showed a soft-tissue mass in the right posterior mediastinum. Enhanced computed tomography scanning showed the mass with similar enhancement to the aortain(white arrow), and the internal density was uneven. **C**, **D**: Coronary reconstruction showed that the mass was connected with the azygos vein(white arrow). **E**: Thoracoscopic surgery revealed a mass arising from the azygos venous arch; there was light adhesion. **F**: The venomous mass was completely removed

**Fig. 2:**
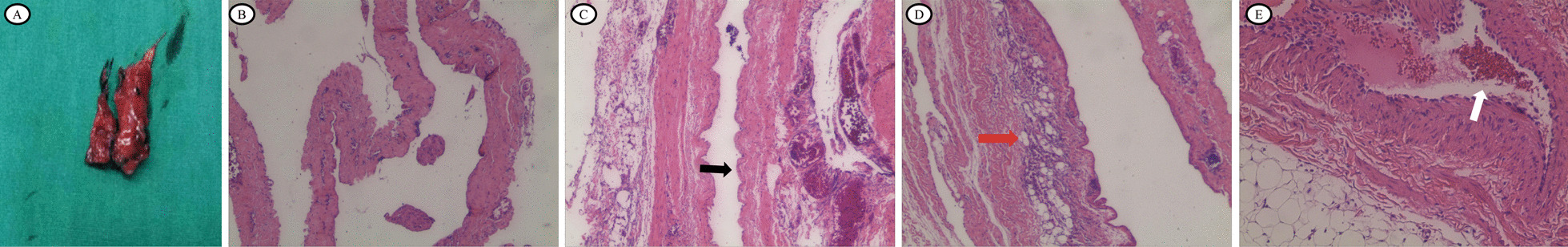
**A**: The removal tissues of saccular azygos vein aneurysm was performed. **B**, **C**: At low magnification, the vascular walls of the hemangioma are distorted and of varying thickness, with the black arrow indicating the thinness of the muscular wall. **D**: Focal fatty vacuoles are seen in the vessel wall (red arrow). **E**: A large number of red blood cells (white arrow) are seen in this partially dilated lumen, and the vessel wall is lined with short spindle endothelial cells

A 37-year-old female was admitted for surgery for sebaceous cyst of the back with no specific underlying disease or symptoms such as dyspnea, cough, chest pain and so on. The pre-operative chest computed tomography revealed a 4.9*3.7*3.2-cm sized well-defined homogenous opacity in the right lower trachea while her physical examination and hematologic test results were normal. Dynamic contrast enhanced multidetector CT of the patient’s chest confirmed a slowly enhancing mass in the pathway of the azygos venous arch. In addition, there seemed to be a blood shadow during coronary reconstruction. The patient was submitted to surgery and thoracoscopic approach to the resection of the tumoral mass by the operation of video-assisted thoracic surgery during which there displayed a mass arising from the azygos venous arch, which was an enlarged abnormally dark purple cystic lesion with slightly adherent to the surrounding tissues (Fig. 1). Finally, en bloc resection of the tumor in the azygos vein arch was safely performed with no intraoperative incidents or accidents. Histopathological examination showed the thin dilated wall lined by intact endothelial cells and thickened medial muscle layer, suggestive of a cystic mass. Meanwhile, a large number of red blood cells were found outside the lumen that formed mural thrombus (Fig. 2). What’s more, postoperative pathological examination confirmed the diagnosis of a hemangioma. The patient was discharged on the seventh postoperative day and there were no abnormal findings 3 months after the operation. After receiving thoracoscopic resection of AVA, the patient did not complain about physical side effects, but rather relieved his concern, which was due to the possible future physical damage caused by AVA. So the patients and their families are very satisfied with the operation.

## Discussion and conclusions

AVA is a greatly rare cause of the posterior mediastinal mass with some symptoms presented depending on the aneurysm size and complications. There have been reports of cases of AVA presenting with some symptoms such as back pain, chest tightness, dyspnea, coughing and orthostatic hypotension [9]. However, complications such as thrombosis [15, 16], rupture, or compression of adjacent organs have been described.

The etiologies of AVA have not been clarified, which are divided into idiopathic, acquired, or traumatic [9] at present. Some scholars speculate that the venous wall is congenitally weak or degenerative due to abnormal connective tissue at the anastomotic site between the proximal segment of the posterior main vein and the right superior main vein in the embryonic stage, and then develops into abnormal formation of idiopathic AVA [17–19]. Connective tissue disorders may represent another congenital cause of AVA formation [20]. Causes of acquired AVA formation that have been proposed include portal hypertension, arteriovenous fistula, cardiac decompensation, pregnancy, and compression of the SVC due to neoplasms or thrombus formation [3, 9, 17]. The acquired AVA is usually fusiform that may easily be influenced by respiratory motion and cardiac pulsation during the imaging process [8]. Traumatic AVA resulting from blunt injury or catheter insertion have been described rarely [9, 21] In our case report, the saccular azygos vein aneurysm is adjacent to the azygos arch while the remaining part of the azygos vein usually appears normal with no specific underlying disease, injury or symptoms. Therefore, we consider a asymptomatic idiopathic saccular AVA, which is surgically resected by thoracoscopic approach and pathologically confirmed.

Azygos vein aneurysms must be taken into consideration during the differential diagnosis of right posterior superior mediastinal masses. In view of the diverse clinical presentation, the diagnosis of azygos vein aneurysms is often confused with lymphomas, bronchial cysts, fibromas, and paragangliomas [5–7, 22]. The key to the differentiation is the relationship between the tumor and the azygos vein, the appearance of contrast agent stratification in enhanced CT, and the consistency of the density/signal between the tumor and the azygos vein in the delayed phase (See Additional file 1 and 2). Furthermore, it should be differentiated from rare tumors such as epithelioid hemangioma and leiomyosarcoma of the azygos vein, which may have malignant biological manifestations such as invasion of adjacent structures. Valuable imaging techniques such as enhanced CT, magnetic resonance imaging, digital subtraction angiography, positron emission tomography/magnetic resonance imaging, and endobronchial ultrasound [2, 3, 10] are the primary modalities employed for initial noninvasive assessments of AVAs, which contributes to clarifying the nature and origin of the lesion through detecting the connection to adjacent vascular structures. But the preoperative diagnosis of azygos vein aneurysms can be very difficult to establish, especially when associated with complete thrombosis that accompany poor enhancementor another thoracic pathology [2, 4, 8, 9]. In the present case, there was a slowly enhancing mass in the pathway of the azygos venous arch confirmed by dynamic contrast enhanced multidetector CT, which showed that the saccular mass was gradually filled with contrast material and three-dimensional reconstruction images revealed a connection to azygos vein arch. Therefore, we made a definitely preoperative diagnosis of an idiopathic sacular azygos vein aneurysms by enhanced multidetector CT. This is one motivation for the reported work.

AVA is a relatively benign condition and its therapeutic strategy is still contentious. At the same time, there are no clear guidelines or therapeutic program for conservative, surgical or endovascular treatment. The aim of any therapy in patients with an AVA is to prevent rupture of the aneurysm, thromboembolism or pulmonary artery hypertension, as well as symptoms caused by compression of adjacent structures there [9, 14]. Yet there is no established conduit or knowledge concerning the optimal therapies for asymptomatic cases. The risk of intraluminal thrombus formation and pulmonary embolism in patients with AVA by conservative observation or oral anticoagulation has been reported [10, 14]. Saccular AVA is typically large that possesses a relatively higher frequency of chest symptoms and intraluminal thrombosis with progressive growth as a result that conservative treatments may be appropriate for patients with fusiform AVA but not fit for saccular AVA. Although there is no literature about azygous vein rupture during conservative treatment that may be related to the low pressure system of azygous vein system, theoretically the risk of rupture increases in larger saccular AVA [9]. Endovascular treatments have also been suggested that some cases reported the successful endovascular treatment of an AVA by embolization of the lumen with coils 12, implantation of covered stents [11], or Amplatzer vascular plugs [23]. However, some scholars think that simple occlusion of the AVA through embolization coils may not prevent future migration and embolization of the thrombus, and treatment with a covered stent or Amplatzer vascular plugs treatment precludes the risk of aneurysm rupture and preserves the patency of the AVA. In fact, a large aneurysm that is filled with numerous coils or Amplatzer vascular plugs may produce worsening of the compressing symptoms by mass effect, and curvature of the AVA may increase the difficulty of stent placement [9, 11, 12, 23]. Surgical resection is a common method that is proposed for cases of idiopathic AVA or symptomatic cases [2, 8, 13–15]. No postoperative recurrence has been reported so far. Surgical treatment should strongly be considered in the presence of clinical chest symptoms or intraluminal thrombosis of saccular AVA, pulmonary embolism or pulmonary arterial hypertension, significant increase in diameter during follow-up or conservative treatment. If it is necessary to remove the aneurysm, video-assisted thoracic surgery may be a good option for surgical treatment. But it must be performed with extreme care in order to prevent intraoperative thrombus migration resulting in pulmonary thromboembolism and massive hemorrhage [2, 13–15]. Although the patient had no clinical symptoms in the study, the idiopathic saccular AVA was relatively larger in diameter and the filling defect was apparently detected by enhanced multidetector CT. Therefore, it might be thrombosis inside AVA. Considering the risk of complications arising from aneurysm such as pressure effects on adjacent structures, venous rupture, and pulmonary embolism, we actively adopted surgical therapy by video-assisted thoracic surgery. The diagnosis of AVA was confirmed by pathological examination. Without intra-operative embolism and no complications during the postoperative, the patient was discharged one week after operation.

In conclusion, AVA is a rare lump-like lesion of the mediastinum, which needs to be included in the differential diagnosis. The imaging findings combined with enhanced multidetector CT and three-dimensional reconstruction are useful in establishing the nature of the mass and the origin of vascular lesions to characterize azygous vein aneurysm. Once AVA with thrombosis or symptoms, surgery is recommended and must be performed. If the preoperative diagnosis might be hard to be established, we should consider avoiding the risk of bleeding caused by puncture biopsy and performing an exploratory thoracoscope.

## Supplementary Information

**Additional file 1**. Contrast-enhanced CT of the patient's chest before surgery, 3D reconstruction of the aortic and azygos vessels.

**Additional file 2**. Non-enhanced CT of the patient's chest before surgery.

## Data Availability

The datasets used and/or analyzed during the current study are available from the corresponding author on reasonable request.

## Abbreviations

AVA: Azygos vein aneurysm
CT: Computed tomography
