# Supplementary figures and images for: Thoracoscopic approach to the resection of idiopathic azygos vein aneurysm: a case report
Source: J Cardiothorac Surg. 2022 Jun 20;17:163. doi: 10.1186/s13019-022-01908-5 (PMC9210694; doi:10.1186/s13019-022-01908-5)

## Slide 1
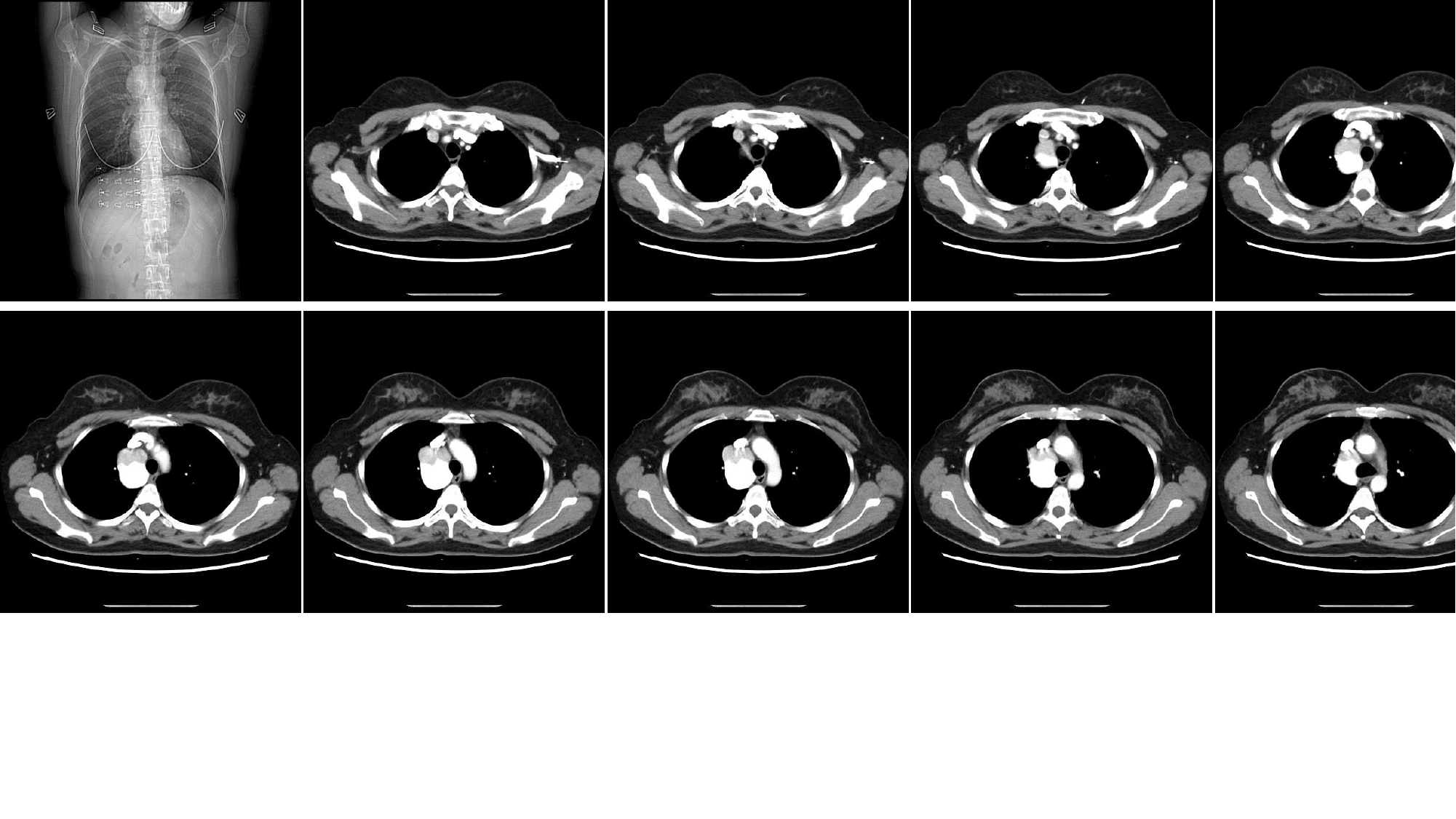

## Slide 2
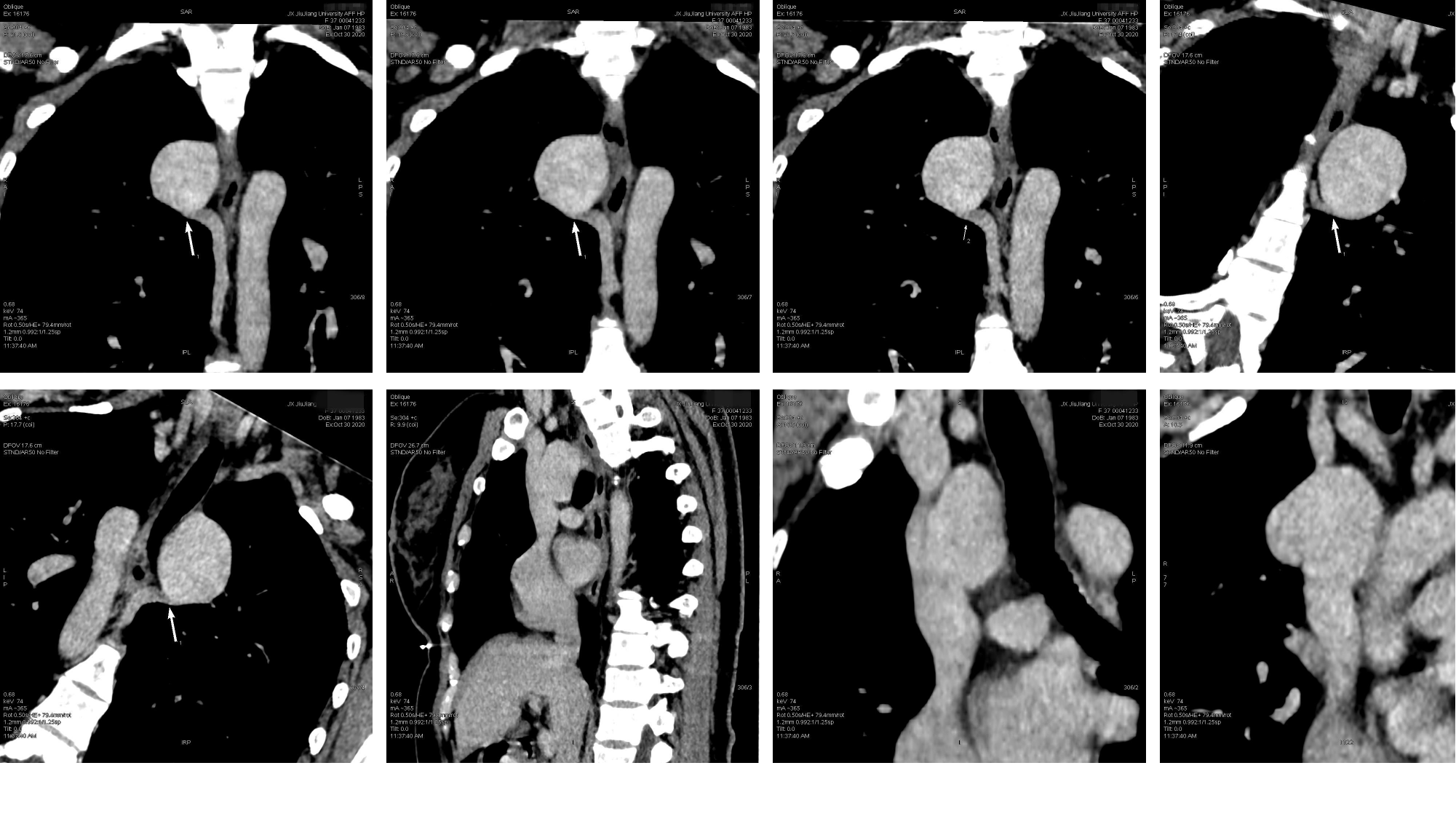

## Slide 3
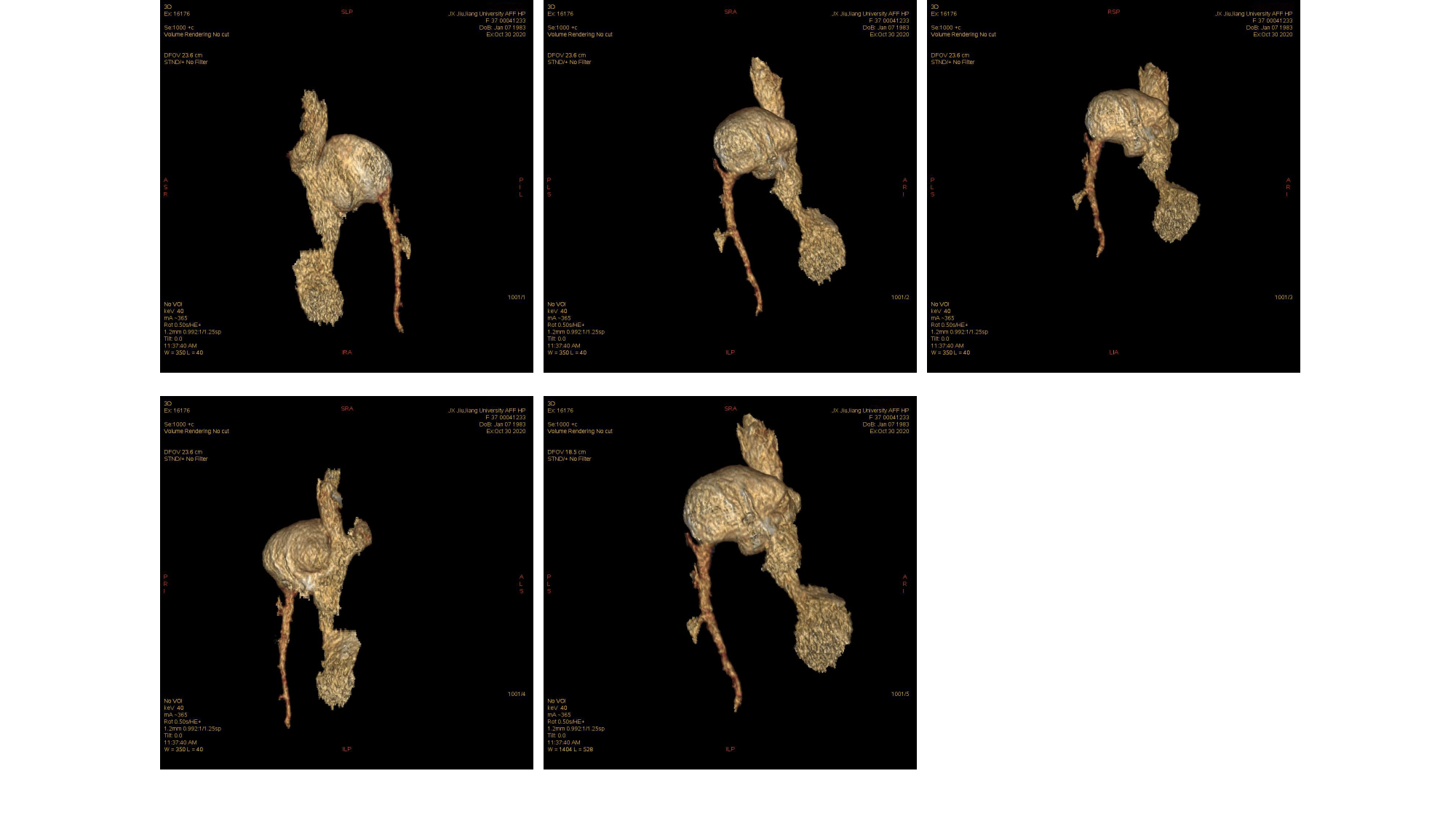

## Slide 4
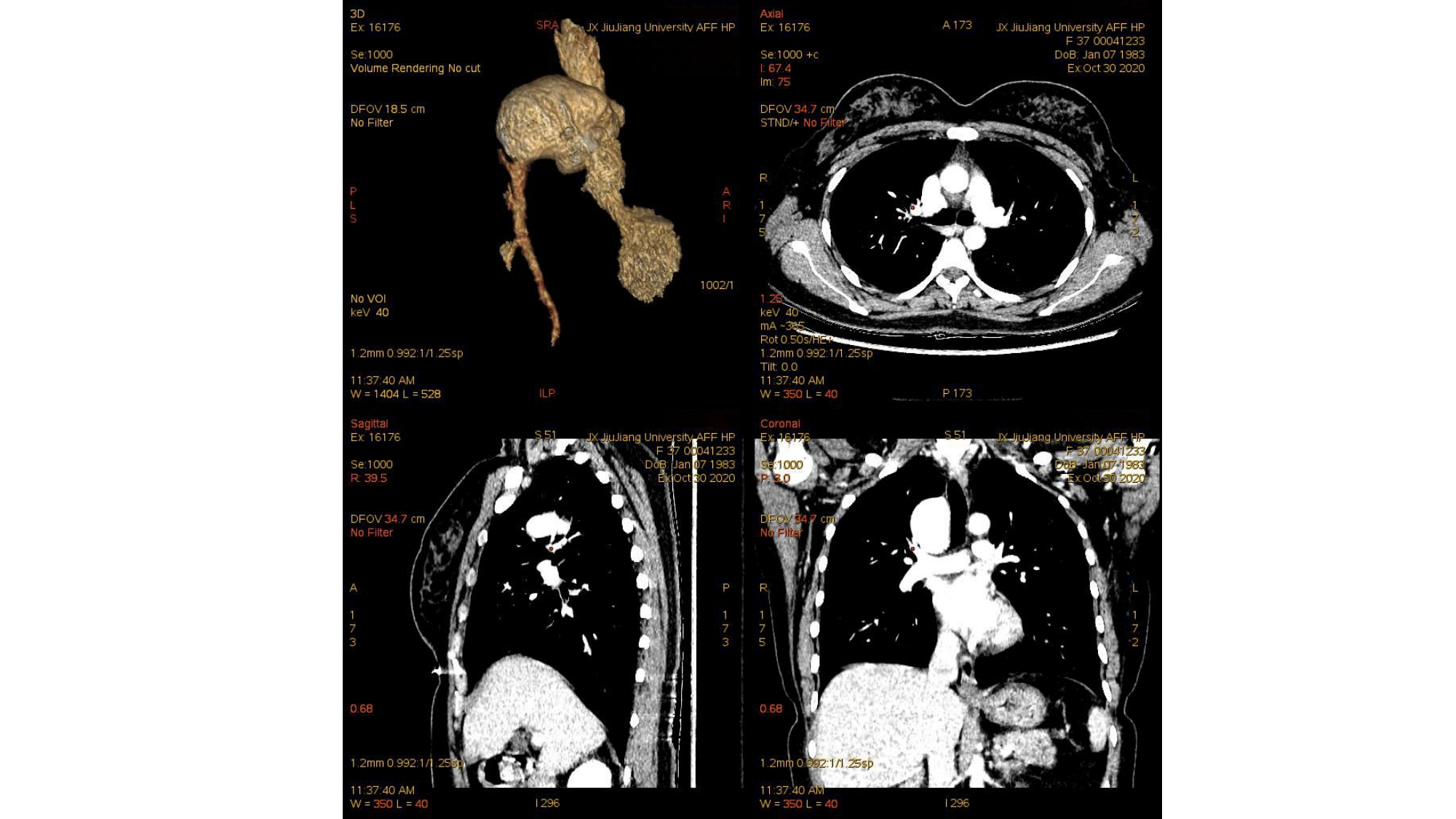

Supplement: Supplementary file 1 — Additional file 1. Contrast-enhanced CT of the patient's chest before surgery, 3D reconstruction of the aortic and azygos vessels. [file 13019_2022_1908_MOESM1_ESM.pptx]

## Slide 1
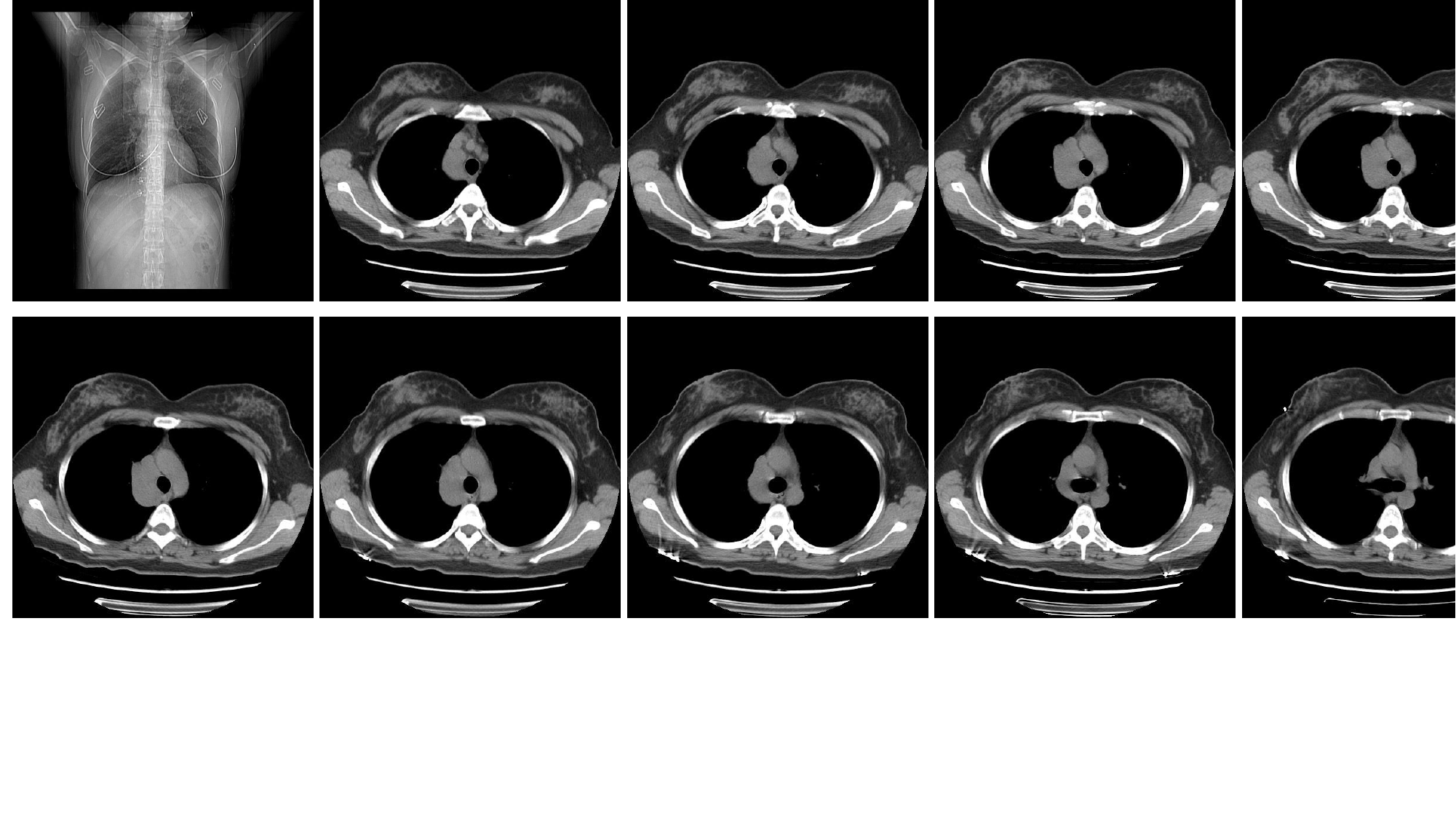

## Slide 2
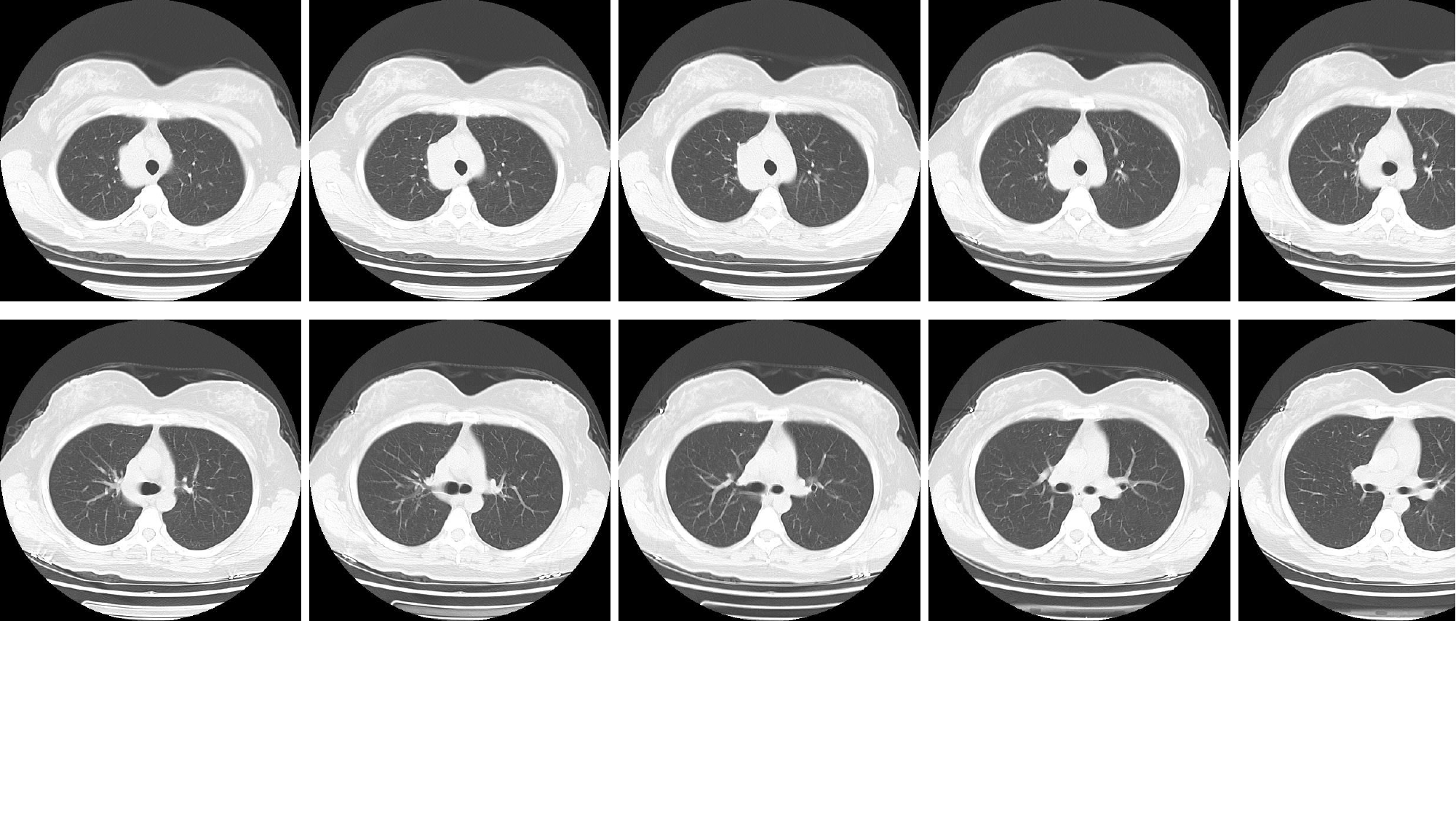

Supplement: Supplementary file 2 — Additional file 2. Non-enhanced CT of the patient's chest before surgery. [file 13019_2022_1908_MOESM2_ESM.pptx]
